# Improvement of T stage precision by integration of surgical and pathological staging in radically resected stage pT3-pT4b gastric cancer

**DOI:** 10.18632/oncotarget.14828

**Published:** 2017-01-26

**Authors:** Hong-Hu Wang, Kai Li, Hao Xu, Zhe Sun, Zhen-Ning Wang, Hui-Mian Xu

**Affiliations:** ^1^ Department of Surgical Oncology, First Affiliated Hospital of China Medical University, Shenyang, Liaoning, China

**Keywords:** surgical staging, pathological staging, prognosis, gastric cancer

## Abstract

**Background:**

Both surgical TNM (sTNM) and pathological TNM (pTNM) staging are important clinicopathologic indexes of gastric cancer (GC). However, surgeons and pathologists might assess tumor depth differently in the same patient. To investigate the prognostic significance of sTNM status in patients with radically resected stage pT3-pT4b GC, we examined the relationship between sTNM and pTNM.

**Methods:**

Clinicopathologic and survival data of 1289 patients with stage pT3-pT4b GC were studied retrospectively, in the aftermath of radical surgery.

**Results:**

The unconformity for assessing tumor invasion depth were frequently exhibited between sT and pT staging. Comparison of 5-year OS among them, no significant differences were observed (pT3/sT3 *vs* pT3/sT4a, *p*=0.962; pT4a/sT4b *vs* pT4b/sT4b, *p*=0.508). Also, pT3/sT4b, pT4a/sT3 and pT4a/sT4a were homogeneity in prognosis. We proposed a revised pT stage in which surgical macroscopic T4b (sT4b) was incorporated into the pT stage, namely, patients in the pT3 stage with sT4b cancers were reclassified as being in the r-pT4a stage; patients in the pT4a stage with sT4b cancers were reclassified as being in the r-pT4b stage. In two-step multivariate analysis, revised pT stage proved more suitable for determining prognosis, surpassing both UICC/AJCC pT stage and sT stage as an independent prognostic index.

**Conclusions:**

Surgical T stage is a significant and independent prognostic index of overall survival (OS) in patients with radically resected advanced GC. Patients in the pT3/4a stage with sT4b cancers, are potentially underestimated, and should be considered higher stage in terms of prognostic.

## INTRODUCTION

Gastric cancer (GC) is a disease with one of the poorest prognoses, and remains the second leading cause of cancer deaths worldwide [1–4]. It is commonly accepted that accurate/optimal staging of primary tumor invasion (pT stage) and regional lymph nodal metastasis (pN stage) is critical for assessing prognosis and decision-making proper adjuvant therapy after curative resection [5–8]. Nonetheless, they are always insufficient for predicting prognosis [9, 10]. Recently, in order to find more suitable prognostic index, many scholars had done a lot and some changes had been done to the pN stage than the pT stage [11–16].

In our previous study, there is another staging system called surgical TNM (sTNM), which is applied to stage and assess in colon and rectum cancer, and it potentially influences patient auxiliary treatment decisions [17]. The sTNM staging is also based on the primary tumors, regional lymph nodes, and metastasis but is defined by surgeons according to the intraoperative findings [18–20]. However, few studies have investigated the prognostic significance of sTNM in gastric cancer. Based on our extensive clinical experience, assessment differences were frequently exhibited between sTNM (macroscopic view) and pathological TNM (pTNM) (microscopic view) staging, especially for patients at T4b stage.

In light of these considerations, the aim of the present study was to investigate prognosis of T3-T4b stage GC patients with inconsistent assessments of tumor invaion between surgical and pathological staging. We also evaluated to integrate the surgical T (sT) stage with pathological T (pT) stage in terms of survival.

## MATERIALS AND METHODS

All patients with gastric cancer who underwent surgery at the Department of Oncology, First Affiliated Hospital of China Medical University from January 1985 to December 2010 were entered into a prospectively maintained database. A total of 1352 patients without distant metastasis or positive peritoneal cytology who underwent curative (R0) surgery (with D2/D3 lymphadenectomy) for primary GC, histologically proven and ultimately were confirmed as stage pT3-pT4b. Of these, 35 died in the postoperative period and were excluded. Follow-up of the entire study population was conducted until cancer related death or the cutoff date (December 31, 2011), by means of outpatient clinic consultation, and/or communication with patients through telephone or letter. Median and mean follow-up period were 29 and 48.55 months (range 1-312 months), respectively. At the time of the last follow-up, 28 were lost and were also excluded, leaving a total of 1289 patients for study enrollment. None received neoadjuvant chemotherapy.

The tumor of each gastric cancer was completely examined, furthermore the final macroscopic depth of invasion was confirmed by all of the surgeons present after tumor exploration. Subsequently the pathologists evaluated the postoperative tumor staging, named pT staging. Macroscopic assessment of tumor depth by surgeons during the operation named sT staging was performed as follows: sT3 lesions were diagnosed when tumor invaded subserosal connective tissue, did not invaded through the serosa; sT4a lesions when serosal involvement were visible and sT4b lesions were tumor invaded the adjacent structures. sT staging was prospectively determined by three surgeons, immediately after the abdominal cavity being opened. Disagreementabout the diagnosis was resolved unanimously after discussion with all three surgeons present.

The study was approved by the Research Ethics Committee of China Medical University. Written informed consent was obtained from all patients prior to participation in this study.

All statistical computations relied on standard software (SPSS version 19.0; IBM Corporation, Armonk, NY, USA). Overall survival rates (OS) were determined by Kaplan-Meier method, using log-rank test was used to identify differences between survival curves of different patient groups. Cox's proportional hazard model (forward-stepwise method) was used for multivariate analysis to identify independent factors predicting prognosis. All *p*-values were two-tailed, with statistical significance set at *p* < 0.05.

## RESULTS

Clinicopathological characteristics of the 1289 patients of this cohort were summarized in Table 1. In our study, there were patients of 748 pT3 (58.03%), 506 pT4a (39.26%) and 35 pT4b (2.72%); but sT3: 238 (18.46%), sT4a: 580 (45.00%); sT4b: 471 (36.54%). Univariate analysis identified age, tumor location, tumor size, UICC/AJCC pT stage, sT stage, UICC/AJCC pN stage, histologic type, lymphovascular invasion and Borrmann type were significantly correlated with prognosis (Table 1).

**Table 1:** Clinicopathologic features of 1289 patients with gastric cancers

Patient Characteristic	*N*^a^	5-YSR(%)^b^	*P*
Gender			0.267
Male	939	34.8	
Female	350	32.7	
Age			0.014
≤ 60 years	697	37.0	
>60 years	592	32.7	
Tumor location			0.000
Entire	95	15.2	
Lower 1/3	766	37.7	
Middle 1/3	226	39.9	
Upper 1/3	202	28.7	
Tumor size			0.000
≤4cm	376	40.5	
>4cm	913	32.8	
UICC/AJCC pT stage			0.000
pT3	748	44.9	
pT4a	506	22.7	
pT4b	35	10.1	
sT stage			0.000
sT3	238	45.9	
sT4a	580	39.4	
sT4b	471	24.8	
UICC/AJCC pN stage			0.000
pN0	348	56.2	
pN1	263	38.4	
pN2	296	29.8	
pN3	382	17.4	
Histologic type			0.001
Differentiated	449	40.0	
Undifferentiated	840	32.5	
Lymphovascular invasion			0.000
Negative	862	39.9	
Positive	358	24.3	
Borrmann type			0.000
Borrmann 1	31	45.2	
Borrmann 2	197	46.7	
Borrmann 3	915	34.4	
Borrmann 4	136	19.5	
Borrmann 5	10	40.0	

Abbreviations: n^a^: Number of patients. 5-YSR^b^: 5-year accumulative survival rate.

**Figure 1:**
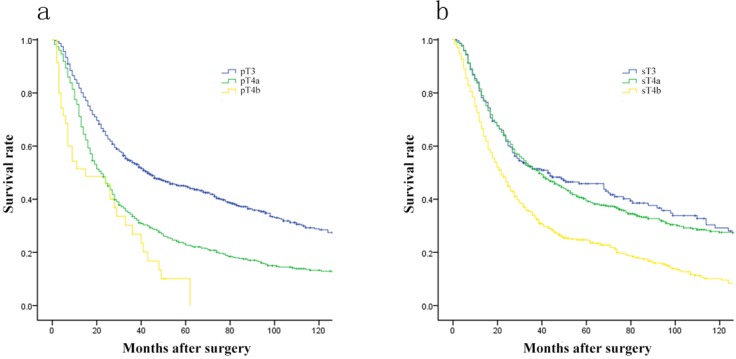
Comparison of survival curves among patients according to the pT and sT stage (each ***p*** = 0.000). **A**., Survival curves of patients with various pT stage. **B**., Survival curves of patients with various sT stage.

**Figure 2:**
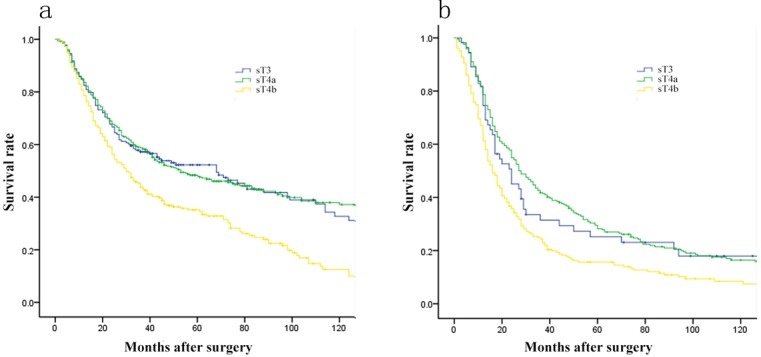
Comparison of survival curves among patients of various sT stage by pT stage **A**. For patients in pT3 stage, there was no prognostic difference between sT3 and sT4a cancer (*p* = 0.962). **B**. For patients in pT4a stage, there was no prognostic difference between sT3 and sT4a cancer (*p* = 0.542).

As shown in Figure 1, prognosis differed significantly among patients of GC in stage pT3-pT4b and sT3-sT4b (each, *p* = 0.000). Comparison 5-year OS in every group of pT stage and sT stage respectively, we found that 5-year OS of patients differed significantly among various pT stages, but not in sT stage (Figure 1B). Subsequently, 5-year OS of various sT stages were compared by pT stage. For pT3 or pT4a stage, there were significant prognostic differences between sT3 and sT4b stage, as well as sT4a and sT4b stage, but not in sT3 and sT4a stage (Figure 2).

**Figure 3:**
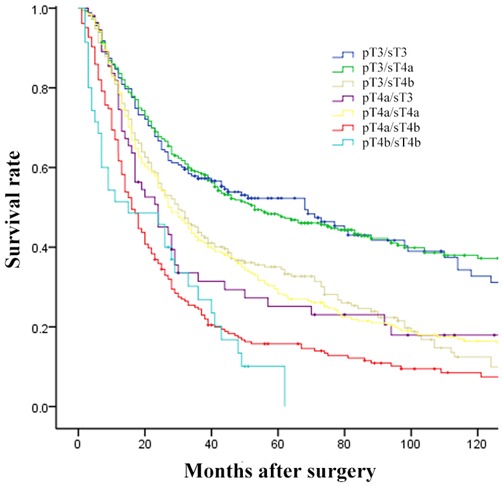
Comparison of survival curves among patients which were divided into 7 groups by pT and sT stage

**Figure 4:**
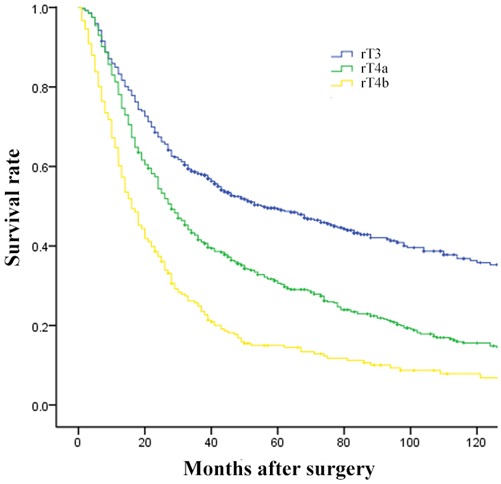
Comparison of survival curves among patients according to the revised T stage ( ***p*** = 0.000).

Furthermore, 1289 patients were divided into 7 groups by pT and sT stage, i) pT3/sT3: 183(14.20%); ii) pT3/sT4a: 336(26.07%); iii) pT3/sT4b: 230(17.84%); iv) pT4a/sT3: 55(4.27%); v) pT4a/sT4a: 244(18.93%); vi) pT 4a/sT4b: 206(15.98%); vii) pT4b/sT4b: 35(2.72%). Comparison of 5-year OS among them, as shown in Figure 3, no significant differences were observed (pT3/sT3 *vs* pT3/sT4a, *p* = 0.962; pT4a/sT4b *vs* pT4b/sT4b, *p* = 0.508), interestingly pT3/sT4b, pT4a/sT3 and pT4a/sT4a was homogeneity in prognosis (pT3/sT4b *vs* pT4a/sT3, *p* = 0.547; pT3/sT4b *vs* pT4a/sT4a, *p* = 0.893; pT4a/sT3 *vs* pT4a/sT4a, *p* = 0.542, respectively)(Table 2). Based on these results, we proposed a revision of pT staging (r-pT), whereby patients at stages pT3/sT4a, pT4a/sT4b were reclassified as stage pT4a, pT4b disease, respectively. Five-year OS curves by pT stage differed significantly for both UICC/AJCC pT staging and r-pT staging (each, *p* = 0.000; Figure 4).

**Table 2:** Comparison of prognosis among patients which were divided into 7 groups by pT and sT stage

VS.	pT3/sT3		pT3/sT4a		pT3/sT4b		pT4a/sT3		pT4a/sT4a		pT4a/sT4b		pT4b/sT4b	
	X^2^	*p*	X^2^	*p*	X^2^	*p*	X^2^	*p*	X^2^	*p*	X^2^	*p*	X^2^	*p*
pT3/sT3			0.002	0.962	17.741	0.000	9.986	0.002	15.967	0.000	57.752	0.000	30.712	0.000
pT3/sT4a	0.002	0.962			25.381	0.000	12.309	0.000	23.791	0.000	83.443	0.000	34.751	0.000
pT3/sT4b	17.741	0.000	25.381	0.000			0.363	0.547	0.018	0.893	19.597	0.000	13.722	0.000
pT4a/sT3	9.986	0.002	12.309	0.000	0.363	0.547			0.371	0.542	4.265	0.037	4.268	0.039
pT4a/sT4a	15.967	0.000	23.791	0.000	0.018	0.893	0.371	0.542			20.017	0.000	12.169	0.000
pT4a/sT4b	57.752	0.000	83.443	0.000	19.597	0.000	4.265	0.037	20.017	0.000			0.439	0.508
pT4b/sT4b	30.712	0.000	34.510	0.000	13.722	0.000	4.268	0.039	12.169	0.000	0.439	0.508		

Univariate analysis identified age, tumor location, tumor size, UICC/AJCC pT stage, sT stage, UICC/AJCC pN stage, histologic type, lymphovascular invasion and Borrmann type were significantly correlated with prognosis (Table 1). To decide which pT staging approach was more suitable as a prognostic index, a two-step multivariate analysis of prognosis in stage pT3-pT4b GC was conducted. In step 1, all significant prognostic factors of univariate analysis were considered, with exception of r-pT stage. We found that age, location of primary tumor, pT stage, sT atsge, pN stage, lymphovascular invasion, and Borrmann type were independent factors in predicting prognosis. However, once r-pT stage was added in step 2, it surpassed both UICC/AJCC pT stage and sT stage as a critical prognostic factor (Table 3).

**Table 3:** Two-step multivariate analysis of prognostic factors for 1289 patients

Characteristics	Multivariate analysis step 1 ^a^			Multivariate analysis step 2 ^B^		
	HR	95%CI	*p*	HR	95%CI	*p*
Age			0.000			0.000
≤ 60 years						
>60 years	1.311	1.148-1.498		1.301	1.139-1.485	
Tumor location			0.000			0.000
Entire						
Lower 1/3	0.679	0.523-0.881		0.683	0.527-0.886	
Middle 1/3	0.679	0.509-0.906		0.684	0.514-0.911	
Upper 1/3	1.037	0.772-1.393		1.057	0.789-1.416	
UICC/AJCC pT stage			0.000			
pT3						
pT4a	1.549	1.350-1.777				
pT4b	1.728	1.109-2.693				
sT stage			0.000			
sT3						
sT4a	0.849	0.697-1035				
sT4b	1.322	1.079-1.619				
UICC/AJCC pN stage			0.000			0.000
pN0						
pN1	1.489	1.213-1.827		1.477	1.204-1.812	
pN2	1.752	1.434-2.140		1.725	1.413-2.105	
pN3	2.597	2.141-3.149		2.569	2.120-3.114	
Lymphovascular invasion			0.002			0.002
Negative						
Positive	1.258	1.088-1.454		1.256	1.087-1.451	
Borrmann type			0.004			0.004
Borrmann 1						
Borrmann 2	0.790	0.497-1.257		0.801	0.504-1.272	
Borrmann 3	1.032	0.665-1.601		1.048	0.676-1.623	
Borrmann 4	1.355	0.836-2.194		1.371	0.847-2.218	
Borrmann 5	0.900	0.358-2.261		0.919	0.367-2.298	
Revised pT stage						0.000
rT3						
rT4a				1.490	1.279-1.736	
rT4b				2.299	1.911-2.767	

Abbreviations: UICC, International Union Against Cancer; AJCC, American Joint Committee on Cancer; HR, hazard ratio; CI, confidence interval.

^a^: Step 1, with consideration of all significantly important prognostic factors in univariate analysis, except for the revised pT stage

^b^: Step 2, with consideration of all significantly important prognostic factors in univariate analysis, including the revised pT stage

## DISCUSSION

In the present study of GC patients in T3-T4b stage, comparison 5-year OS in every group of pT stage and sT stage respectively, we found that survival of patients differed significantly among various pT stage, and also in sT stage. In the next step, 1289 patients were divided into 7 groups by pT and sT stage. Our data revealed that there were no significant prognostic differences between pT4a/sT3-4a and pT3/sT4b, as well as pT4a/sT4b and pT4b/sT4b. Based on these results, we therefore formulated our r-pT staging, where pT3/sT4b and pT4a/sT4b stages GC were incorporated as stage pT4a and pT4b disease, respectively. In this setting, univariate analysis revealed that both sT and pT were important independent prognostic factors. Two-step multivariate analysis confirmed that r-pT stage surpassed both UICC/AJCC pT stage and sT stage as a critical prognostic factor in the prognostic hierarchy.

Our previous study explained some possible reasons of uncomformity between pT stage and sT stage [19, 21]. Major present study additions to this line of investigation were as follows: (1) our study focused on stage T3-T4b GC, because it was difficult to perform accurate assessment of stage T1-T2 GC during the operation; (2) patients were divided into 7 subgroups by pT and sT stage, furthermore were grouped into 3 groups in terms of prognosis; (3) we proposed the revised T stage, importantly integrated macroscopic tumor invasion into TNM stage.

Although primary tumor invasion is significantly and independently predictive of 5-year OS in GC patients, UICC/AJCC pT stage remains always insufficient for predicting prognosis, and recently many scholars have reported that some novel T stages based on several indexes was more suitable in predicting survival [10, 14, 22, 23]. According to numerous previous studies and our practice, the unconformity for assessing tumor invasion depth were frequently exhibited between sT and pT staging [21]. Our finding revealed that there were 835 patients with inconsistent depth of invasion in this setting, therein 436 patients were T4b stage. Pathologists and surgeons have respective difficulty in determining whether tumor invaded the adjacent structures or not. It may be the cause that pathologists and surgeons lack for enough intraoperative finding and sufficient histologic proof during operation, respectively.

The integration of sT and pT staging has distinct advantages and alluring prospects in clinical application [17]. Macroscopic T stage is easily assigned by surgeons, based on gross serosal appearances during open laparotomy, providing a simple and effective method to assess patient prognosis [14, 24]. Likewise, microscopic T stage is routinely reported by pathologists after operation. We may use the macroscopic and microscopic integration to infer the most accurate depth of primary tumor infiltration, thus avoiding a migration in postoperative pT stage. In consideration of the prognostic value of macroscopic and microscopic integration in our hands, we think that its status is critical in deciding whether and how intraoperative and postoperative adjuvant therapy is administered. Thus, further studies are needed to test this premise.

Several limitations of this investigation are acknowledged. The surgical T stage mainly depends on gross macroscopic observations, which may partially vary by individual. Also, This retrospective study (*n* = 1289) was confined to a single Chinese institution. Larger international multicenter samplings should be analyzed to confirm our findings.

In conclusion, our data demonstrate that both surgical T stage and pathological T stage are independent factors for predicting prognosis in patients with radically resected stage pT3-pT4b GC. Importantly, their integration could be applied to more accurately predict the patient prognosis.
